# Vestibular exercise for adjunctive therapy in the management of non-motor functions among Parkinson's patients: A review

**DOI:** 10.6026/973206300213197

**Published:** 2025-09-30

**Authors:** Edagottu Annaiah, Deepika Chandrasekaran, Sai Sailesh Kumar Goothy, Jabir P.K., Sendil Kumaran D.

**Affiliations:** 1 Department of Physiology, Meenakshi Academy of Higher Education and Research (MAHER), Tamil Nadu, India; 2 Department of Physiology, MMCHRI, MAHER, Kanchipuram, Tamil Nadu, India; 3 Department of Physiology, BGS Medical College and Hospital, Bengaluru, Karnataka, India; 4 Department of Physiology, Saveetha Medical College, SIMATS, Chennai, Tamil Nadu, India; 5 Department of Physiology, SMSIMSR, Chikkaballapur, Karnataka, India

**Keywords:** Insomnia, autonomic dysfunction, cognitive decline, neuro-degenerative disorders, non-pharmacological therapies

## Abstract

Non motor symptoms in Parkinson disease are present in about 88% of patients and they include different symptoms like insomnia, autonomic dysfunction, cognitive decline etc. The natures of neurodegenerative process and non-dopaminergic neuropathological changes are yet to be identified. Vestibular and visual deficit are non-studied symptoms in Parkinson's disease. Vestibular rehabilitation exercises are known to improve the function and coordination of the vestibular system, thereby enhancing balance, reducing dizziness and improving spatial awareness. Additionally, vestibulospinal reflex exercises offer better static and dynamic firmness and functional symmetry in daily life situations. Thus, we show the evidence map for management of non-motor symptoms of Parkinson's disease through vestibular stimulation.

## Background:

Parkinson disease (PD) is neurodegenerative disorder, a 2nd most common condition characterized by tremor, rigidity, abnormal gait, bradykinesia and disturbance in non-motor functions like depression, cognitive impairment, anxiety, hyposmia and constipation [1]. Parkinson's disease is believed to be a dopamine insufficient disorder, but in present days evidences suggest that it is a multisystem disease of brain which involves different and numerous brain areas which includes bulb, brainstem, cerebral cortex, olfactory and hypothalamus [2]. Recent study evidence, says that, longstanding clinical descriptions, non-motor symptoms exist, even much before motor symptoms develop and are recognized [3]. The non-motor symptoms are thought to further deteriorate the patient's condition and causes decline in the quality of life. Non motor symptoms in Parkinson disease is present in about 88% of patients and they include different symptoms like - Neuropsychiatric conditions - depression, anxiety, panic attacks, cognitive dysfunction - dementia, hallucinations, psychosis. Disorder of sleep wake cycle - insomnia, restless leg syndrome, autonomic dysfunctions like - CV dysfunction, fatigue. GI dysfunction like constipation, fetal incontinence, Genitourinary dysfunctions are urinary urgency, incontinence, sexual dysfunction, sweating, pain and sensory dysfunction, joint pains, visceral pains. These symptoms can be present at point or stage of the disease. The reasons for these non-motors symptoms are multifactorial but it is said that the reason could be nature of neurodegenerative process and non-dopaminergic neuropathological changes because of the disease [4]. Vestibular and the visual deficit are the few of the least informative and non-studied symptoms [5-6]. Vestibular deficits in Parkinson disease are identified and they include impaired vestibular-ocular reflex, hypofunctions of vestibular [7- 8]. By assimilating the assessment of vestibular and direct therapeutic methods into clinical practice, clinicians can deliver more nuanced and complete care for patients with Parkinson disease [9]. The Parkinson disease affects approximately 1-2% of population, ≥ 65 years of age and around in 3-5% over the age of 85 years [10-11]. The frequency of this disease is increasing sharply with increase in age [12]. Parkinson's disease affects both sexes, predominantly in males, particularly with growing age and mostly in western populations. Diagnosis of the disease is based on clinical presentation; though, it does require identification of both dopaminergic neuron loss and Lewy bodies [13]. Hence, in this review article, we aimed to study vestibular exercise as an adjunctive therapy in the management of non-motor functions in Parkinson's patients. Therefore, it is of interest to review the gap between identifying vestibular dysfunction and its effective management in PD.

## Parkinson's disease and non-motor symptom:

The Non-motor symptoms in Parkinson's patients are a range of symptoms which are not directly related to movement. It includes mood disorders like depression and anxiety, cognitive impairment, pain and fatigue, sleep disturbances, loss of smell, autonomic dysfunction. These symptoms affect mental, sensory and bodily functions away from physical movement difficulties which are associated with Parkinson's disease [14]. Although the cause is unknown, it is believed that there could be interface between genetic susceptibility, environmental factors and age-related processes. The etiopathology of Parkinson disease is that there is a loss of dopaminergic neurons at specific selective region, from the par's compact of substantia niagra. However, evidences show that it is a multi-system disease of brain which compromises of motor and non-motor symptoms.

## Onset of symptoms and diagnosis:

Onset process of the disease is gradual and slow, symptoms often go unnoticed for a long time. When diagnosis is done, patients usually recall signs and symptoms that happened a decade before. Initially, symptoms are seen as loss of dexterity or foot drag [13]. Early symptoms can be flexion of one arm, lack of arm swing, monotonous speech and stiff facial expressions. Early loss of smell is also commonly reported by patients and often goes unnoticed [12]. Though there is emphasis of research on motor related symptoms, there is no much importance on non-motor symptoms. Recently the research studies suggest that non-motor symptoms with Parkinson disease have a larger implication, for quality of life, institutionalization and health economics. With increase in the age and life expectancy among PD, non-motor symptoms become more important. Non-motor symptoms are autonomic dysfunction, sleep disturbances, olfactory deficits, gastrointestinal dysfunction, sensory complaints and neuropsychiatric problems including cognitive impairment, vision changes like blurred vision, diplopia and fatigue [15]. Regrettably, non-motor symptoms are ignored despite their significance. It is believed that for the development of non-motor symptoms, non-dopaminergic-cell dysfunction plays a major role. While, the neuroanatomical and neurochemical substrates remain unknown.

## Results:

A part of non-motor symptoms is not been studied in oculo-visual and vestibular problems [16, 17]. Diverse oculo-visual difficulties have been recognized in Parkinson disease comprising decreased color, static visual acuity deficits and contrast sensitivity, depth perception deficits, eye movement deficits which is understudied and under-recognized (Table 1 - see PDF) [18]. Vestibular deficits contain abnormal vestibular-ocular reflex and vestibular hypofunction which can be unilateral, bilateral and central [5]. Vestibular deficits are significant in Parkinson disease for the reason that of balance and postural stability, influence on overall function and quality of life [6]. Parkinson disease patients commonly report with impaired visual function, difficulty in reading regardless of normal visual acuity testing. It was Nowacka *et al.* [7] said that there was reduced distance and near best modified visual acuity when compared to age-matched controls. Integrated Visual/Vestibular control is important for gaze stabilization which is used for reading, head movements and overall stability of posture. Impaired balance is distinguishing feature among Parkinson disease people, particularly when disorder progresses. This affects nearly 75% of those who are diagnosed with PD. Repeated falls are reported among 50% of such individuals [18]. Undeniably, in PD, the contributors for impaired balance have gained attention which includes loss of postural reflexes, irregular use of muscles of lower extremity, loss of mobility of axial spine [19]. Other examples for physiological contributors are impaired balance and falls among older adults which include weakness in lower extremity, vision loss and proprioceptive loss [20, 21]. Many studies have reported the evidences for the insufficiencies in vestibule-ocular (VORs) and vestibule-spinal reflexes [22- 23]. It is been believable that shortfalls can be appeared in vestibulo spinal reflexes with or without VOR being evident [24]. There has been great advancement in recent years in technology for detection of vestibular deficits. It is decided that in few of the studies, vestibular dysfunction was existent but not able to detect.

## Vestibular exercises [27, 28-29]:

The vestibular system located within inner ear, plays a very critical role in maintaining the balance and spatial orientation. It provides sensory information to the brain about head position, movement and changes in gravitational forces. In Parkinson's disease, the dysfunction of the vestibular system can contribute to balance problems and increase the risk of falls. Vestibular rehabilitation exercises aim to improve the function and coordination of the vestibular system, thereby enhancing balance, reducing dizziness and improving spatial awareness. These exercises typically involve head movements, eye exercises and activities that challenge balance and coordination. The vestibulo-ocular reflex stimulation, encourages variations in neuronal response to head movements, allows restored alignment of head and contributes to postural balance function (Table 2 - see PDF). Additionally, vestibulospinal reflex exercises offer better static and dynamic firmness and functional symmetry in daily life situations [30].

## Gaze stabilization exercises:

These exercises focus on maintaining a stable gaze while moving the head (05-10min). They help improve visual tracking and reduce dizziness or vertigo symptoms.

## Balance training:

Various exercises (05-10min) can challenge balance, like standing on a leg, tandem walking-heel-to-toe, or performing balance exercises on unstable surfaces like foam pads or balance boards.

## Eye-head coordination exercises (05-10min):

These exercises involve coordinating eye movements with head movements, such as tracking objects with the eyes while moving the head side to side or up and down.

## Head Tilts:

Sit or stand with good posture and slowly incline head to one side, taking your ear closer to your shoulder. Alternate between left and right sides, keeping the movements controlled and smooth [31].

## Tandem Walking:

Walk along straight line, keeping one foot in front directly of the other (heel-to-toe). Focus on maintaining balance and a stable gaze as you walk slowly and deliberately [32].

## Modified Sit-to-Stand:

Practice standing up from a seated position while keeping your gaze fixed on a specific point in front of you. This exercise combines vestibular input with balance and coordination.

## Balancing on One Leg:

Stand near a stable support (*e.g.*, a chair) and lift one leg off the ground. Maintain your balance while keeping a stable gaze. Hold the position for a few seconds and then switch legs. Certain studies conducted effectiveness of vestibular exercises among Parkinson disease and found that it helps to improve postural balance improved performance in oculomotor function of fixation and movements. Postural stability, dynamic balance and static exercises are being proposed in vestibular rehabilitation session, which will help to contribute to posture adjustment performance [34]. Vestibular therapy is specified for cases where patients suffer from posture imbalance, irrespective of disease staging. It is said that the posture instability demonstrates greater intensity from stage III onwards, which can elicit recurrent episodes of imbalance in posture. Vestibular rehabilitation has great prospective to contribute to postural balance among patients with Parkinson's disease.

## Treatments available for non-motor functions in Parkinson's disease:

Before clinician starts with medical treatment for Parkinson's disease, they recommend patients to visit physical therapist, speech and occupational therapist. The goal of such consultation helps to manage altered motor symptoms. An important feature for rehabilitation is regular exercise. There is lot of options available for the treatment of motor symptoms, while therapy options for the treatment of non-motor symptoms are insufficient. As Parkinson's can be considered as neuropsychiatric disorder, its symptoms are related to emotional and cognitive issues. This disorder significantly disrupts and leads to disability in patients with Parkinson disease. Managing non-motor symptoms of Parkinson disease offers a challenge to physician as they should be able to differentiate the involvement from medication, disorder progression and their emotional state. Most of the drugs that are approved by US FDA for the treatment of non-motor symptoms help to treat depression, anxiety, gastrointestinal issues and excessive drooling. But a single drug prescribe will not be able to effectively treat each of this non-motor symptoms as the symptoms often are known to arise from different underlying mechanism from the brain or body [35]. Vestibular stimulation has been stated to advance the multi-sensory processing. Vestibular Stimulation is known to progress posture stability among elderly patients. It has also been stated that incorporation of vestibular signals with sensory signals helps in maintaining postural stability. The vestibular system has integration with medulla, midbrain and spinal cord to maintain postural balance [36]. In bio behavioral research, a well-accepted result deals is quality of life (QOL) of patients. It has been appeared that measuring QOL is reliable for testing effectiveness. Particularly, non-motor symptoms of Parkinson disease worsen QOL of patients. This includes alteration in the appetite, lack of sleep psychological distress. Vestibular stimulation increases physical fitness by improving muscular activity. In another way, the vestibular stimulation is increased by physical health by relaxing effect and increases sleep. Psychological well-being is improved by increase blood flow to brain, thereby improving brain metabolism, which influence frontal cortex and acetylcholine secretion in brain. Vestibular stimulation enhances mood, behavior by cortisol and increases social relationships by increasing physical, psychological and social relationships, it rises environmental domain as well [32]. Hence, Vestibular rehabilitation should be planned as per signs and symptoms shown by every patient, irrespective of peripheral and central findings of the vestibular evaluation. Though there is very little evidence regarding the application in Parkinson disease patients, establishing its effects on postural imbalance is necessary for improved prognosis [33].

## Vestibular exercise as adjuvant therapy in Parkinson disease:

Though vestibular system is associated with dysfunction which is prevalent, its underlying mechanism is not completely understood. Its pathologic, physiological and anatomical evidence supports the impression that Parkinson disease patients have vestibular dysfunction. Use of medications for Parkinson disease treatment can cause serios adverse effects, which may limit its use. Noninvasive vestibular exercise therapy is now associated with better compliance than pharmacotherapy, because of effectiveness. Hence it is recommended in US clinical practice guidelines [37]. Vestibular exercise therapy helps to improve majority of the symptoms related to Parkinson disease. This therapy works via repetition of exact physical exercise, which wills active the central neuroplastic mechanism in order to attain adaptive benefit of impaired function. Balance training intensifies thickness of cortical in vestibular cortical regions, which helps vestibular compensation. There is an increase in the evidence that has established positive effects of vestibular exercise therapy in Parkinson disease patients who experience dizziness and balances the disturbance, like augmenting vision and proprioception to recompensate the loss, to develop strategies for imbalance, to develop substitution for assisting gaze stability. Virtual realism and few technologies will advance and become more regularly functional in VR therapy, contributing endowed clinical applications [38].

## Conclusion:

Vestibular exercise therapy helps to improve majority of the symptoms related to Parkinson disease. Though the underlying mechanism of vestibular dysfunction is not clearly understood, the targeted treatment related to vestibular symptoms may increase Parkinson disease symptoms and improve quality of life. Further numerous investigations are required which will have implications beyond the symptoms in Parkinson disease and other clinical conditions. Future studies can consider this as preliminary point and can focus on evaluating if targeted treatment of vestibular system can support to delay the progression of Parkinson disease and can consider vestibular exercise therapy as adjuvant to Parkinson disease therapy.

## Funding:

Self-funding.

## Ethical Approval:

Approved by the IEC (IEC/ certificate/39/2024 dated 21-06-2024).

## Abbreviations:

VR= Virtual Realism Therapy, PD= Parkinson's disease.
